# Detection of SARS-CoV-2 Genome in Stool and Plasma Samples of Laboratory Confirmed Iranian COVID-19 Patients

**DOI:** 10.3389/fmolb.2022.865129

**Published:** 2022-06-28

**Authors:** Mobin Makhmalbaf, Seyed Masoud Hosseini, Hamid Asadzadeh Aghdaei, Mahsa Saeedi Niasar, Shahrzad Shoraka, Abbas Yadegar, Shaghayegh Baradaran Ghavami, Shabnam Shahrokh, Mohammadreza Moshari, Habib Malekpour, Mohammad Reza Zali, Seyed Reza Mohebbi

**Affiliations:** ^1^ Basic and Molecular Epidemiology of Gastrointestinal Disorders Research Center, Research Institute for Gastroenterology and Liver Diseases, Shahid Beheshti University of Medical Sciences, Tehran, Iran; ^2^ Department of Microbiology and Microbial Biotechnology, Faculty of Life Sciences and Biotechnology, Shahid Beheshti University, Tehran, Iran; ^3^ Gastroenterology and Liver Diseases Research Center, Research Institute for Gastroenterology and Liver Diseases, Shahid Beheshti University of Medical Sciences, Tehran, Iran; ^4^ Foodborne and Waterborne Diseases Research Center, Research Institute for Gastroenterology and Liver Diseases, Shahid Beheshti University of Medical Sciences, Tehran, Iran; ^5^ Department of Anesthesiology, Faculty of Medicine, Shahid Beheshti University of Medical Sciences, Tehran, Iran; ^6^ Research and Development Center, Imam Hossein Hospital, Shahid Beheshti University of Medical Sciences, Tehran, Iran

**Keywords:** SARS-CoV-2, COVID-19, RT-qPCR, plasma, feces, COVID-19 nucleic acid testing

## Abstract

Coronavirus disease 2019 (COVID19), caused by the severe acute respiratory syndrome coronavirus 2 (SARSCoV2), was first discovered in China in late 2019 and quickly spread worldwide. Although nasopharyngeal swab sampling is still the most popular approach identify SARS-CoV-2 carriers, other body samples may reveal the virus genome, indicating the potential for virus transmission *via* non-respiratory samples. In this study, researchers looked at the presence and degree of SARS-CoV-2 genome in stool and plasma samples from 191 Iranian COVID-19 patients, and looked for a link between these results and the severity of their disease. SARS-CoV-2 RNA shedding in feces and plasma of COVID-19 patients was assessed by reverse transcription-quantitative polymerase chain reaction (RT-qPCR). Medical data were collected and evaluated, including Clinical features, demographics, radiological, and laboratory findings of the patients. Plasma samples from 117 confirmed laboratory patients were evaluated and 24 out of 117 patients (20.51%) tested positive for SARS-COV-2 RNA. Besides, 20 out of 74 patients (27.03%) tested positive for SARS-COV-2 RNA in stool samples. There seems to be no relationship between the presence of SARS-CoV-2 genome in fecal and plasma samples of Covid-19 patients and the severity of illness. We provide evidence of the SARS-CoV-2 genome presence in stool and plasma samples of Iranian COVID-19 patients.

## 1 Introduction

In late December 2019, a series of strange pneumonia cases were reported in China, with symptoms that were strikingly similar to viral pneumonia, and soon spread to other parts of the globe (Zhu et al., 2020). The Chinese Center for Disease Control and Prevention (CDC) found a novel coronavirus in a patient’s throat sample in early January 2020, and the World Health Organization (WHO) named it SARS-COV-2 (Cheung et al., 2020). Before SARS-CoV-2, two other outbreaks were reported by two other members of the coronavirus family, occurred in 2002 by SARS-CoV and in 2012 by MERS-CoV, respectively (Lippi et al., 2020). The SARS-CoV-2, which belongs to the lineage B of the genus Beta coronaviruses, has a single-stranded RNA-positive genome with about 30,000 nucleotides length and is very similar to the SARS-CoV (Wu et al., 2020). But unlike the previous two outbreaks, SARS-CoV-2 has caused millions of deaths in terms of severe respiratory complications, mainly dyspnea and other organ failures (Lippi et al., 2020). In addition to the usual symptoms of respiratory diseases, other symptoms such as chest pain, headache, and especially gastrointestinal symptoms are also observed in patients. The most common clinical indications of SARS-COV-2 infection are vague, and many of these symptoms may also be caused by other viruses that cause respiratory tract infections (Udugama et al., 2020).

On the other hand, in the current pandemic, efficient diagnostic methods are especially needed and help control the prevalence of the virus. Covid-19 patients are currently diagnosed using molecular and serological methods. Unlike molecular methods, serological methods have low accuracy in the early detection of patients with Covid-19 (Dhamad and Abdal Rhida, 2020). RT-qPCR is standard gold technique among all diagnostic methods. In diagnosing patients with SARS-CoV-2 infection, the combination of pharyngeal RT-qPCR and chest CT is more sensitive than other methods (Ren et al., 2020; Wei et al., 2020; Zhang et al., 2021).Several studies on the plasma of Covid-19 patients have also found that the amount of SARS-2 plasma RNA in these patients is directly related to the severity of the disease. These studies emphasize that examining the presence of SARS CoV-2 RNA and its viral load in plasma samples of patients with Covid-19 helps to predict prognosis of the disease (Veyer et al., 2020).Therefore, Covid-19 patients’ plasma analysis in terms of the presence of SARS Cov-2 RNA can be effective in better management of the covid-19 pandemic (Thijssen et al., 2020; Colagrossi et al., 2021). Furthermore, regarding the high presence of the SARS-CoV-2 genome in feces, to improve the diagnosis of the carrier, it is better to use fecal RT-qPCR as a case for hospital discharge (Ren et al., 2020; Wang et al., 2020). Several studies have confirmed the presence of the SARS-CoV-2 genome in the fecal samples of patients with Covid-19, suggesting that oral-fecal transmission of the virus can occur (Chen et al., 2020a).

Moreover, this research aimed to investigate the presence of the SARS-CoV-2 genome in the stool and plasma of Iranian patients with Covid-19, and its correlation to clinical symptoms.

## 2 Methods

### 2.1 Study Design and Patients

The enrolled patients were admitted to Taleghani Educational Hospital and Imam Hossein Educational Hospital, Shahid Beheshti University of Medical Sciences from April 6th to 15 November 2020. The study protocols were approved by the ethics committee of the Research Institute for Gastroenterology and Liver Disease (IR.SBMU.RIGLD.REC.1399.007, Tehran, Iran), and informed consent was collected from all participants.

All patients in this study were confirmed to have Covid-19 due to a positive nasopharyngeal RT-qPCR test and symptoms of pneumonia on a chest Computed tomography (CT) scan. Patients who did not have Covid-19 laboratory confirmed were not included in the study. It is noteworthy that the RT-PCR cycling threshold (Ct) for determining whether people are positive or negative for Covid-19 is a CT ≤ 40. Therefore, individuals with a CT > 40 were excluded from the study.

### 2.2 Sample Collection

All patients were admitted to the hospital on average 8–10 days from onset of symptoms. Fecal samples were collected from 74 registered COVID-19 patients at the beginning of admission and on the second and fourth days of the clinical course up to 3 times serially. Plasma samples were also collected from 117 other registered COVID-19 patients with the same conditions. Then all samples were stored in a special container in the freezer at −20°C. The volume of samples collected in each turn for fecal samples is between 10 and 20 ml and for plasma samples is between 5 and 10 ml.

### 2.3 Data Collection

191 patients demographic information, clinical characteristics (including symptoms, medical history, underlying diseases), and epidemiological, laboratory results of all patients were collected from Taleghani Educational Hospital and Imam Hossein Educational Hospital, SBMU using medical information registration system and evaluated individually.

### 2.4 Laboratory Assays

Plasma and feces samples were obtained and analyzed for SARS-CoV-2 RNA using RT-qPCR during the hospitalization period. Laboratory confirmation of SARS‐CoV‐2 was performed by Gastroenterology and Liver Diseases Research Center of Shahid Beheshti University of Medical Sciences in Taleghani Hospital. The nucleic acid extraction from the stool and plasma samples was performed using QIAamp Viral RNA Kit following the manufacturer’s instructions (QIAGEN, Germany). Extracted nucleic acid specimens were evaluated for SARS-CoV-2 with RT-qPCR using a SARS-CoV-2 ORF1ab/N Gene Nucleic acid detection kit (Sansure, China) and the Rotor-Gene Q real-time PCR system (QIAGEN, Germany) under manufacturer’s instructions; 4 μl 2019-nCoV-PCR-Enzyme Mix were added into 26 μl of the 2019-nCoV-PCR Mix. Then add 30 µl PCR-Master mix into PCR reaction tube with 20 µl processed sample. Reactions were incubated at 50°C for 30 min and 95°C for 1 min followed by 45 cycles at 95°C for 15 s and 60°C for the 30 s.

The severity of COVID-19 was assessed by WHO interim recommendations (World Health Organization, 2020).

### 2.5 Statistical Analysis

GraphPad Prism v.8.4.1 (GraphPad Software, San Diego, CA, United States) was used for all statistical analyses. The data distribution was statistically normal, according to Shapiro-Wilks test. As well as, homogeneity of variance was significant (*p < 0.05*)*,* underlying that the presumption of the normality or homogeneity was met for given specimens. Therefore, student’s t-test and χ2 analysis were used to test for statistical differences among patients with negative and positive RT-qPCR tests, with a two-sided *p*-value of 0.05 or lower signifying significance.

## 3 Results

### 3.1 Demographics and Clinical Characteristics

From April 6th to 15 November 2020, from the beginning of the Covid-19 pandemic to the end of the third wave of this epidemic, a total of 191 COVID‐19 hospitalized patients at Taleghani and Imam Hossein Educational Hospital, SBMU, were enrolled. Patients’ demographic and basic characteristics that were studied are listed in Table 1.

**TABLE 1:** Baseline characteristics of all 117 patients.

Symptoms	Patients with plasma samples (*n* = 117)	Patients with stool samples (*n* = 74)
Fever	42 (35.89%)	29 (39.18%)
Dry Cough	46 (39.31%)	29 (39.18%)
Dyspnea	55 (47.00%)	33 (44.59%)
Myalgia	23 (19.65%)	18 (24.32%)
Headache	8 (6.83%)	4 (5.40%)
Chest pain	11 (9.40%)	6 (8.1%)
Diarrhea	9 (7.69%)	6 (8.1%)
Nausea	19 (16.23%)	15 (20.27%)
Anorexia	17 (14.52%)	9 (12.16%)
Underlying diseases	67 (52.26%)	42 (56.75%)
Men	77 (65.81%)	44 (59.45%)
Women	40 (34.18%)	30 (40.54%)
age (mean ± SD)	58.13 ± 17.84	55.12 ± 15.94

### 3.2 Presence of SARS-COV-2 Genome in Plasma Samples

Plasma samples of patients were collected to identify SARS-CoV-2 RNA multiple times during the hospitalization stage. As shown in Table 2, 24 of 117 (20.51%) patients analyzed positive for SARS‐CoV‐2 RNA in plasma. 12 patients were positive in the first sample (2 weeks after the onset of symptoms), four were positive in the second sample (day 3 of admission) and five patients were positive at the third sample (day 5 of admission), and other 92 (78.63%) patients tested negative. Moreover, the vital signs of patients such as heart rate, respiratory rate, temperature, and blood pressure, were recorded. 61.44 and 40.96% of patients were positive for the presence of IgG and IgM in plasma, respectively.

**TABLE 2:** A comparison of demographic and clinical data of patients with SARS-CoV-2 RNA positive/negative results in plasma samples.

Symptoms	All patients (*n* = 117)	RNA (-) in plasma (*n* = 92)	RNA (+) in plasma (*n* = 24)	*p* value
Fever	42 (35.89%)	30 (30.60%)	11 (45.83%)	0.487
Dry Cough	46 (39.31%)	34 (36.95%)	12 (50%)	0.328
Dyspnea	55 (47.00%)	43 (46.73%)	12 (50%)	0.965
Myalgia	23 (19.65%)	16 (17.39%)	7 (29.16%)	0.346
Headache	8 (6.83%)	6 (6.52%)	2 (8.33%)	0.724
Chest pain	11 (9.40%)	9 (9.78%)	2 (8.33%)	0.841
Diarrhea	9 (7.69%)	8 (33.33%)	1 (1.08%)	0.678
Nausea	19 (16.23%)	15 (16.30%)	4 (16.66%)	0.591
Anorexia	17 (14.52%)	15 (16.30%)	2 (8.33%)	0.338
Underlying diseases	67 (52.26%)	51 (55.43%)	16 (66.66%)	0.351
Men	77 (65.81%)	57 (61.95%)	20 (83.33%)	0.42
Women	40 (34.18%)	36 (39.13%)	4 (16.66%)	0.42
age (mean ± SD)	58.13 ± 17.84	59.29 ± 18.00	53.65 ± 16.85	0.178

Data are median (IQR), n (%), or n/N (%), where N is the total number of COVID‐19, patients with available data. *p* values comparing the patients who tested SARS‐CoV‐2 RNA (+) and SARS‐CoV‐2 RNA (−) in plasma are from χ^2^ test, Fisher’s exact test, or Mann‐Whitney *U* test.

COVID‐19, coronavirus disease 2019; RNA (+), positive for RNA; RNA (−), negative for RNA.

*p* < 0.05 was considered statistically significant.

The median (IQR) age was 58.13 ± 17.84 (18–90) years, and 40 (34.18%) of these were women. 67 (52.26%) patients had underlying diseases, including diabetes, kidney disease, cardiovascular disease, hypertension, chronic pulmonary disease, chronic kidney disease, liver disease, and cancer. The most common clinical symptoms among patients were shortness of breath (55, 47.00%), dry cough (46, 39.31%), fever (42, 35.89%), Myalgia (23.19.65%), and nausea (19, 21.56%). Less common symptoms were headache (8.6.83%), Diarrhea (9.7.69%), Chest pain (11.9.40%). Remarkably, 12 (50.00%) patients with fever and dyspnea and 16 (66.66%) patients with underlying disease tested positive for plasma RT-PCR. Also, 20 (83.33%) patients who tested positive for plasma RT-qPCR were men.

### 3.3 Presence of SARS-COV-2 Genome in Stool Samples

Stool samples from 74 individuals were collected and examined for the presence of SARS-COV-2 genomic RNA. Eighteen patients tested positive on the first day of admission, two patients tested positive on the third day of admission, and one patient tested positive on the fourth day of hospitalization (day 5 of admission). Table 3 shows the findings of this investigation, as well as clinical and demographic information about these individuals.

**TABLE 3:** A comparison of demographic and clinical data of patients with SARS-CoV-2 RNA positive/negative results in stool samples.

Symptoms	All patients (*n* = 74)	RNA (-) in feces (*n* = 53)	RNA (+) in feces (*n* = 21)	*p* value
Fever	29 (39.18%)	17 (32.07%)	12 (57.14%)	0.063
Cough	29 (39.18%)	17 (32.07%)	12 (57.14%)	0.063
Dyspnea	33 (44.59%)	20 (37.73%)	13 (61.90%)	0.078
Myalgia	18 (24.32%)	10 (18.86%)	8 (38.09%)	0.115
Headache	4 (5.40%)	2 (3.77%)	2 (9.5%)	0.375
Chest pain	6 (8.1%)	4 (7.54%)	2 (9.5%)	0.867
Diarrhea	6 (8.1%)	5 (9.4%)	1 (4.76%)	0.44
Nausea	15 (20.27%)	12 (22.64%)	3 (14.28%)	0.308
Anorexia	9 (12.16%)	6 (11.32%)	3 (14.28%)	0.871
Underlying diseases	42 (56.75%)	31 (58.49%)	11 (52.38%)	0.24
Men	44 (59.45%)	33 (62.26%)	11 (52.38%)	0.634
Women	30 (40.54%)	21 (39.62%)	9 (42.85%)	
Age (mean ± SD)	55.12 ± 15.94	58.74 ± 15.82	46.6 ± 12.93	0.004

Data are median (IQR), n (%), or n/N (%), where N is the total number of COVID‐19, patients with available data. *p* values comparing the patients who tested SARS‐CoV‐2 RNA (+) and SARS‐CoV‐2 RNA (−) in plasma are from the χ^2^ test, Fisher’s exact test, or Mann‐Whitney *U* test.

COVID-19, coronavirus disease 2019; RNA (+), positive for RNA; RNA (−), negative for RNA.

*p* < 0.05 was considered statistically significant.

44 (59.45%) of these patients were male, 30 (40.54%) were female and the mean age of these was 55.12 ± 15.94 (24–86) years. 42 (56.75%) patients had an underlying disease, including diabetes, kidney disease, cardiovascular disease, hypertension, chronic pulmonary disease, chronic kidney disease, liver disease, and cancer.

Less common symptoms were headache (4.5.40%), chest pain, and diarrhea (6.8.1%). The most common clinical symptoms among patients were fever (6.42.85%), anorexia (9.12.16%), nausea (15.20.27%). Stool samples were collected to detect COVID-19 RNA several times during the hospitalization stage. As shown in Table 3, 21 of 74 (28.37%) patients tested positive for SARS‐CoV‐2 RNA in feces, and the other 53 (71.62%) patients tested negative. It is important to note that all those who had nausea tested positive for SARS‐CoV‐2 RNA RT-PCR (*p* value *=* 0.308).

### 3.4 Laboratory and Vital Finding

The laboratory and vital data of all patients whose plasma samples were evaluated for the presence of SARS COV-2 RNA are recorded in Table 4. Compared to people whose plasma SARS‐CoV‐2 RNA RT-qPCR test results are negative, the average amount of Na (*p* value = 0.333) and d-dimer (*p* value = 0.270) in the blood of people whose plasma SARS‐CoV‐2 RNA RT-qPCR test results are positive are higher, and this difference is not significant.

**TABLE 4:** Laboratory Parameters of COVID‐19 patients with plasma on admission.

Parameters	All SARS CoV-2 patients (mean ± SD) (*n* = 117)	RNA (-) in plasma (mean ± SD) (*n* = 92)	RNA (+) in plasma (mean ± SD) (*n* = 24)	*p* Value
Systolic BP	124.35 ± 20.25	124.98 ± 20.43	122.22 ± 19.94	0.577
Diastolic BP	77.38 ± 12.23	77.33 ± 1,185	77.52	0.951
Heart Rate bpm	86.14 ± 13.47	85.13 ± 12.68	89.47 ± 15.67	0.197
Respiratory Rate rpm	17.80 ± 1.54	17.66 ± 1.55	18.16 ± 1.52	0.343
Temperature	37.06 ± 0.85	36.98 ± 0.73	37.29 ± 1.18	0.173
O2saturation	90.93 ± 4.63	90.68 ± 4.81	91.78 ± 3.93	0.336
WBC× 109/L	8.79 ± 5.12	8.64 ± 4.44	9.32 ± 7.22	0.585
lymphocyte	17.79 ± 8.70	18.21 ± 8.78	16.35 ± 8.43	0.392
PLT× 109/L	229.16 ± 112.54	224.13 ± 110.49	247.08 ± 120.36	0.390
ALT U/L	34.46 ± 21.22	34.47 ± 21.56	34.43 ± 20.61	0.995
AST U/L	40.14 ± 27.99	40.30 ± 29.82	39.59 ± 21.64	0.918
Hb g/ml	11.32 ± 2.32	11.17 ± 2.37	11.82 ± 2.08	0.273
Na mEQ/L	138.68 ± 5.18	138.93 ± 5.45	137.72 ± 3.97	0.333
K mEQ/L	4.08 ± 0.62	4.04 ± 0.62	4.21 ± 0.60	0.253
Cr mg/dl	1.26 ± 0.65	1.27 ± 0.68	1.21 ± 0.55	0.687
LDH	618.71 ± 335.89	601.13 ± 287.87	673.52 ± 461.52	0.433
ESR	38.53 ± 26.404	40.01 ± 27.96	33.71 ± 20.38	0.342
D-dimer mg/L	784.39 ± 973.49	681.55 ± 866.03	1,017.46 ± 1,182.14	0.270
CRP	21.33 ± 19.96	23.75 ± 21.18	11.29 ± 8.66	0.024

Note: Data are median (IQR), n (%), or n/N (%), where N is the total number of COVID‐19, patients with available data. *p* values comparing the patients who tested SARS‐CoV‐2 RNA (+) and SARS‐CoV‐2 RNA (−) in feces are from χ^2^ test, Fisher’s exact test, or Mann‐Whitney *U* test.

COVID‐19, coronavirus disease 2019; SARS‐CoV‐2, severe acute respiratory syndrome coronavirus two; RNA (+), positive for RNA; RNA (−), negative for RNA. *p* < 0.05 was considered statistically significant.

Besides, laboratory findings and vital data of patients whose stools sample were evaluated for the presence of SARS‐CoV‐2 RNA by RT-qPCR test, are shown in Table 5, which showed the mean temperature of patients whose stool samples were positive for SARS-CoV-2 RNA was higher than that of patients whose stool samples were negative for SARS-CoV-2 RNA, and this difference was significant.

**TABLE 5:** Laboratory Parameters of COVID‐19 patients with feces on admission.

Parameters (mean)	All patients (mean ± SD) (*n* = 74)	RNA (-) in feces (mean ± SD) (*n* = 53)	RNA (+) in feces (mean ± SD) (*n* = 21)	*p* value
Systolic BP	125.37 ± 19.00	125.97 ± 20.88	123.87 ± 13.66	0.712
Diastolic BP	77.17 ± 12.19	78.18 ± 12.74	74.66 ± 13.41	0.378
Heart Rate bpm	87.94 ± 14.43	89.13 ± 14.12	84.61 ± 15.35	0.338
Respiratory Rate rpm	17.80 ± 1.54	17.66 ± 1.55	18.16 ± 1.52	0.343
Temperature °c	37.08 ± 0.74	36.87 ± 0.50	37.63 ± 0.98	0.002
O2 saturation	91.86 ± 3.68	92.00 ± 3.11	91.51 ± 4.97	0.692
WBC× 109/L	8.38 ± 4.89	8.97 ± 5.36	7.02 ± 3.32	0.172
Lymphocyte	18.29 ± 7.35	18.60 ± 7.89	17.47 ± 5.97	0.632
PLT× 109/L	232.09 ± 121.74	247.40 ± 136.61	193.81 ± 70.98	0.138
ALT U/L	37.09 ± 23.81	36.81 ± 24.31	37.62 ± 23.57	0.911
AST U/L	45.02 ± 37.54	50.63 ± 43.49	33.42 ± 16.21	0.134
Hb g/ml	10.96 ± 2.56	10.73 ± 2.64	11.54 ± 2.33	0.336
Na mEQ/L	139.16 ± 5.22	139.47 ± 5.86	138.31 ± 2.98	0.453
K mEQ/L	4.04 ± 0.54	4.03 ± 0.59	4.05 ± 0.40	0.922
Cr mg/dl	1.33 ± 0.84	1.31 ± 0.82	1.36 ± 0.89	0.854
LDH	673.49 ± 365.38	687.92 ± 412.19	643.41 ± 254.51	0.734
ESR	35.50 ± 24.29	35.12 ± 25.79	36.33 ± 21.42	0.875
D-dimer	1,457.40 ± 1790.86	1,673.00 ± 1863.33	1,275.84 ± 1757.42	0.506
CRP	9.58 ± 6.38	9.96 ± 7.10	8.75 ± 4.61	0.591

Note: Data are median (IQR), n (%), or n/N (%), where N is the total number of COVID‐19, patients with available data. *p* values comparing the patients who tested SARS‐CoV‐2 RNA (+) and SARS‐CoV‐2 RNA (−) in feces are from χ^2^ test, Fisher’s exact test, or Mann‐Whitney *U* test.

COVID‐19, coronavirus disease 2019; SARS‐CoV‐2, severe acute respiratory syndrome coronavirus two; RNA (+), positive for RNA; RNA (−), negative for RNA. *p* < 0.05 was considered statistically significant.

### 3.5 Correlation Between Disease Severity and Viral Load in Stool and Plasma Samples

Figure 2 indicates the severity of COVID-19. In general, 44 (37.60%) of patients whose plasma samples were examined, belonged to the severe clinical group. 20% of Severe patients were positive for the presence of RNA virus in plasma samples. Furthermore, 27 (36.48%) of the patients whose stool samples were analyzed and classified as severe, with 46.66% testing positive for an RNA virus.

**FIGURE 1:**
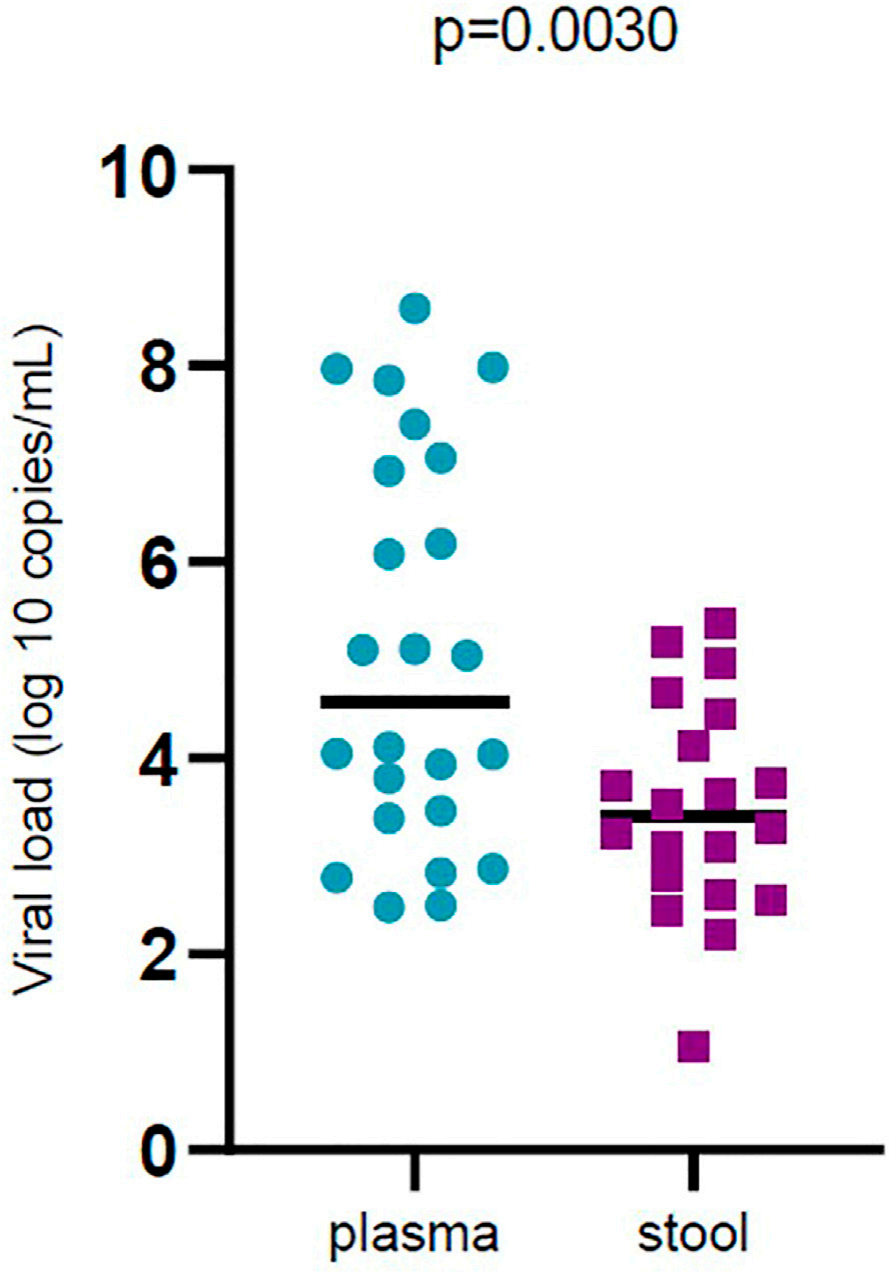
Comparison of severe acute respiratory syndrome coronavirus 2 (SARS -CoV-2) viral load by sample types (plasma and stool). Black bars represent medians.

**FIGURE 2:**
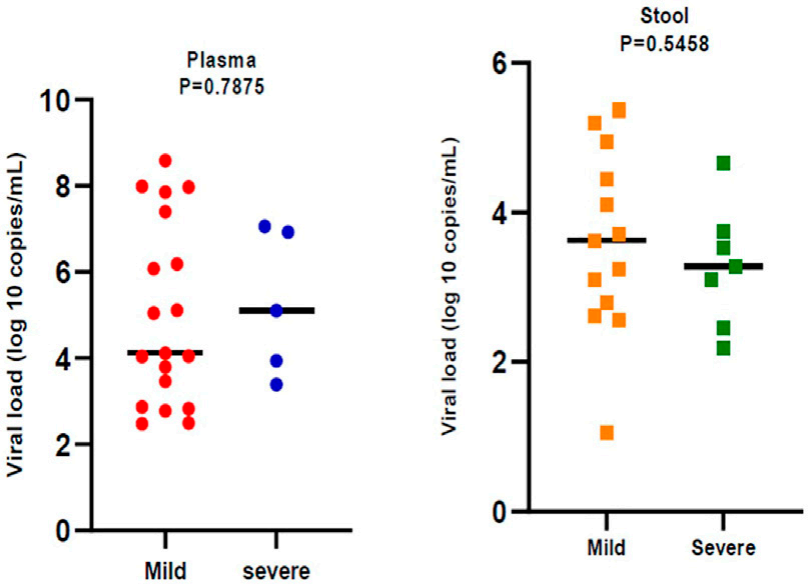
Comparison of severe acute respiratory syndrome coronavirus 2 (SARS -CoV-2) viral load by disease severity. Black bars represent medians.

In general, 28.37% of fecal samples and 20.51% of plasma samples were positive for SARS‐CoV‐2 RNA and their viral load was evaluated. Viral load was significantly different (*p* < 0.05) in plasma samples compared to stool samples. Plasma samples showed higher viral load than stool samples (Figure 1). Viral loads in plasma and fecal samples did not show a significant difference among the patients with mild disease and patients with severe disease (Figure 2).

## 4 Discussion

The lungs are primary SARS-CoV-2’s target organ. The respiratory system is also recognized to be the virus’s most prevalent route of transmission. SARS-CoV-2 penetrates target cells by attaching to the ACE-2 receptor, and since this receptor is found on the surface of other organ cells as well as lung cells, there’s a chance it might infect cells other than lung cells. Therefore, transmission routes other than the respiratory route should also be considered (Robba et al., 2020; Trypsteen et al., 2020; Vinayagam and Sattu, 2020). However, using multiple samples at the same time could prevent probable viral transmission through other routes like the oral-fecal and other body fluids (Wang et al., 2020). Moreover, RNA shedding in different biological samples and recognizing its transmission routes in terms of biosafety is essential for health care workers and patient management in hospitals (Eastin and Eastin, 2020). Furthermore, for a more accurate diagnosis of Covid-19 patients and reduce viral shedding, it is necessary to analyze the association between the severity of illness and the presence of the virus in different samples of patients.

The presence of pathogens in various samples of other viruses was considered. For example, Hepatitis C virus RNA, has been found in the saliva, bile, and feces of chronic hepatitis C patients. Patients with a higher serum HCV viral load had more positive saliva samples. HCV RNA was reported to be higher in the feces of patients with low platelets, in addition to the presence of the virus genome in the feces of men more than women. (Yanaga et al., 1997; Suzuki et al., 2005; Wang et al., 2006; Heidrich et al., 2016). Furthermore, in studies on the Ebola virus, genomic RNA was found in a variety of bodily fluids, including urine, feces, blood, sweat, saliva, tears, cerebrospinal fluid, amniotic fluid, breast milk, and sperm, in acute Ebola virus disease patients (Rodriguez et al., 1999; Bausch et al., 2007; Feldmann and Geisbert, 2011; Sagui et al., 2015; INTERIM GUIDANCE, 2016). In a study, it was stated that the viral load in the feces of Ebola patients peaked during the disease (Prescott et al., 2015; Schibler et al., 2015). Also in a study conducted on plasma, urine, and saliva samples of patients with dengue virus by Andris et al., 85.4% of plasma samples, 41.6% of urine samples, and 39% of saliva samples in terms of The presence of dengue virus genome were positive (Andries et al., 2015). According to a study by Chan et al., SARS-CoV RNA is detected only in feces after day 5, and the proportion of positive stool samples increases until day eleven when it reaches its peak (Chan et al., 2004).

The presence of the viral genome in different clinical samples is highly important and its relationship with the severity of clinical symptoms, this study was designated to investigate the presence of SARS-CoV-2 genome in stool and plasma samples of patients with Covid-19 and to study their association with clinical signs of individuals. Thus, our result showed stool samples from 74 patients and plasma samples from 117 patients with Covid-19 were evaluated. 27.03% of stool samples and 20.51% of plasma specimens were positive for the SARS-CoV-2 genome.

Many diagnostic procedures have been explored to detect infected patients since the commencement of the Covid-19 outbreak. Molecular assays for the identification of the SARS-CoV-2 genome or serological testing for the presence of antibodies to the virus are used to identify Covid-19 patients (Jarrom et al., 2020). Methods such as RT-qPCR, which identifies the viral nucleic acid, are frequently used to detect the presence of the virus. For the clinical diagnosis of SARS-CoV-2, the RT-qPCR of the nasopharyngeal swab and chest CT scan is commonly used. The sensitivity of RT-qPCR tests to identify Covid-19 patients was found to be 88% in research by Burnheim et al. (Bernheim et al., 2020). The RT-qPCR test was negative in a percentage of individuals whose Covid-19 was approved, according to Lee et al. (Li et al., 2020a). This might be due to a sample deficit, a laboratory mistake, or a lack of virus particles in the sample Regarding the prevalence of COVID-19 and the high potential of SARS-CoV-2 transmission *via* non-respiratory routes, also due to the high viral load in these samples, studying different samples for viral genome to reduce virus prevalence is significant.

Considering the importance of the issues raised, in research on samples from laboratory-confirmed COVID-19 patients, Chen et al. found that 88.2 percent of pharyngeal specimens, 11.5% of plasma specimens, and 21.2% of fecal specimens were positive for SARS-CoV-2 RNA (Chan et al., 2020). In another research, 59% of stool samples, 41% of plasma samples, and 1% of urine samples were positive for the COVID-19. The viral load of respiratory samples was stronger than in other samples, according to Zheng et al. (Jarrom et al., 2020). Lamers et al. Have presented evidence of contamination and proliferation of SARS-Cov-2 in small intestinal enterocytes, which increases the likelihood of infection and proliferation of these particles in human intestinal enterocytes (Lamers et al., 2020).Wu et al. (Bernheim et al., 2020) showed that the SARS-COV-2 RNA was present in the laboratory-confirmed COVID-19 patients’ fecal samples to 5 weeks after the negative nasopharyngeal test. Although no confirmation of SARS-CoV-2 particles being transmitted through feces has been found, several studies have raised concerns about the shedding of viral active particles through patients’ feces (Lamers et al., 2020; Mirjalali et al., 2020; Tian et al., 2020).

Chou et al. stated in a research on the transmission of SARS-CoV-2 via blood products that the likelihood of transmission through this route should be examined (Cho et al., 2020). Meanwhile, the European Center for Disease Control and Prevention (ECDC) suggests delaying blood donations for 21 days after any probable contact with authorized patients in order to prevent the virus from spreading *via* blood products (Risk Assessment, 2020). As a result, the role of blood products in the transmission of the SARS-COV-2 is important.

Although it is critical to assess non-respiratory samples such as stools and blood to prevent the virus from spreading in pandemics and to ensure the safety of healthcare personnel, it is critical to examine the relationship among clinical signs or laboratory data and the presence of the SARS-CoV-2 genome in various samples.

For example, Chen et al. Found that The severity of COVID-19 pneumonia was positively correlated with plasma CRP levels that a non-specific reactive protein that increased in infection and inflammation. High levels of this protein have been observed in the plasma of Covid-19 patients, which can be used for diagnosis and prognosis (Chen et al., 2020b). In our study, people who had a plasma positive for SARS-CoV-2 RNA had a higher CRP than people who did not have SARS-CoV-2 RNA. This can increase the possibility of the presence of an active virus and can also be used to diagnose and prognosis.

As well, because fever in patients is a sign of active immunity (Moltz, 1993; Young and Saxena, 2014), and according to this study’s findings, the temperature in patients whose SARS-CoV-2 RNA was detected in their feces is higher than patients whose feces are negative for SARS-CoV-2 RNA, the possibility of SARS-CoV-2 active particles exists in these patients is increased.

There is debate as to whether SARS-CoV-2 shedding in stool and plasma samples is associated with the severity of the disease. Some studies found no link, which is consistent with the findings of our research (Chen et al., 2020a). Other research has shown a link between SARS-CoV-2 shedding in stool and plasma samples and the severity of COVID-19 symptoms in humans (Bermejo-Martin et al., 2020; Li et al., 2020b; Reuken et al., 2020; Shang et al., 2020).

No association was found between disease severity and gastrointestinal symptoms with the presence of SARS-CoV-2 RNA and the possible presence of the virus in the fecal and plasma samples of Covid-19 patients. This difference among studies may be in terms of environmental and genetic differences affecting clinical signs in the course of the disease, and differences in terms of SARS-CoV-2 strains.

Our study was performed on limited patients. more specimens better define viral shedding in plasma and feces and their association with clinical symptoms, and the usefulness of recommending routine testing of non-respiratory specimens.

The deterministic correlation between plasma and fecal viral shedding and the severity of gastrointestinal symptoms has not yet been determined, but it appears that SARS-CoV-2 may be present in the gut without affecting the severity of gastrointestinal symptoms.

Although SARS-CoV-2 RNA was detected in the feces of Covid-19 patients, further studies are needed to prove or disprove its infectious potential.

## Data Availability

The raw data supporting the conclusions of this article will be made available by the authors, without undue reservation.
